# From career calling to proactivity in healthcare professionals: the roles of responsibility, mission commitment, and responsible leadership

**DOI:** 10.3389/fpsyg.2026.1789838

**Published:** 2026-04-10

**Authors:** Jianyue Chen, Jiajia Ren

**Affiliations:** 1 Academy of Civil Aviation Transportation, Chengdu Aeronautic Polytechnic University, Chengdu, China; 2 XuZhou Central Hospital, Xuzhou, China

**Keywords:** career calling, mission commitment, proactive work behavior, responsible leadership, sense of responsibility

## Abstract

Against the backdrop of recurrent public health risks and continuously rising demands for healthcare services, healthcare professionals increasingly perform under conditions characterized by heavy workloads and high uncertainty. In such contexts, proactive work behavior is pivotal for improving service quality, safeguarding patient safety, and enhancing organizational resilience. Career calling—defined as a deep-seated sense of occupational meaning, value, and prosocial purpose—may motivate healthcare professionals to engage in proactivity that goes beyond formal role requirements. Drawing on role identity theory and social information processing theory, we develop and test a moderated mediation model in which career calling promotes proactive work behavior through two psychological mechanisms—sense of responsibility and mission commitment—and in which responsible leadership amplifies these effects. Survey data from 417 healthcare professionals were analyzed to evaluate the proposed model. Results show that (a) career calling is positively associated with proactive work behavior; (b) sense of responsibility and mission commitment each mediate the relationship between career calling and proactive work behavior; (c) responsible leadership strengthens the positive effects of career calling on sense of responsibility and mission commitment; and (d) responsible leadership further intensifies the indirect effects of career calling on proactive work behavior via both mediators, providing evidence for moderated mediation.

## 1 Introduction

In an external environment marked by escalating uncertainty and complexity, healthcare organizations face multiple structural pressures, including the recurrent disruption of public health events, heightened societal expectations regarding service quality and patient safety, and rising levels of burnout and turnover intention among healthcare professionals (Zhang et al., 2012; Cutler et al., 2022; Kajaria-Montag et al., 2024; Lai et al., 2024). Under these conditions, healthcare professionals must not only deliver high-intensity work that is strongly constrained by professional standards, but also sustain performance and safety under resource scarcity, role conflict, and emotional depletion (Gaines and Jermier, 1983). Consistent with the job demands–resources (JD–R) framework, high demands elevate exhaustion risk, whereas insufficient resources undermine individuals' capacity for self-regulation and sustained work engagement, thereby producing a chronic health-impairment process (Demerouti et al., 2001).

Within this high-demand/low-resource context, proactive work behavior becomes particularly consequential. Proactivity entails more than performing prescribed duties; it involves anticipating problems, initiating improvements, and enacting constructive changes that extend beyond formal role boundaries to enhance organizational functioning and service quality (Morrison and Phelps, 1999). Prior work suggests that proactivity enables organizations to respond more rapidly to external demands, correct process deficiencies, and build resilience in uncertain environments—fundamentally reflecting future-oriented, self-initiated improvement (Griffin and Grote, 2020; Morrison and Phelps, 1999; Rindova and Courtney, 2020). Yet, in healthcare settings characterized by strong institutional constraints and limited slack resources, proactivity is rarely “free.” Healthcare professionals often must weigh whether and how to act proactively against time pressure, heightened accountability exposure, and potential interpersonal and career risks. Accordingly, a central question for organizational behavior and human resource management scholarship is how healthcare organizations can sustainably elicit and maintain proactive work behavior among healthcare professionals.

A particularly relevant lens for addressing this question is career calling (Elangovan et al., 2010; Xie et al., 2016; Schabram et al., 2023). Career calling typically refers to viewing one's work as deeply meaningful and socially valuable, accompanied by identity-based commitment and sustained investment that transcends narrow self-interest (Praskova et al., 2015). Theory and reviews further suggest that calling is not merely a positive orientation, but often contains a normative component—an “ought-to” sense of moral obligation—such that individuals preferentially allocate effort toward goals perceived as worthwhile and necessary (Dobrow et al., 2023; Dobrow and Tosti-Kharas, 2011). This value orientation is especially salient in healthcare, where work is inherently public-facing and prosocial; calling may provide a durable meaning foundation and moral impetus that sustains effort and discretionary contributions under high workload.

Despite this promise, existing research has not fully explained why and when calling translates into proactive work behavior. The calling literature has largely emphasized affective and attitudinal outcomes (e.g., well-being, work meaning, occupational commitment) or has examined boundary effects through broad forms of external support (e.g., perceived organizational support), leaving relatively limited integrative theorizing regarding the more proximal, behavior-relevant psychological pathways through which calling promotes proactive behavior (Eisenberger et al., 1986; Parker et al., 2006; Dobrow et al., 2023; Xie et al., 2026). This gap is notable because proactivity typically requires at least two psychological conditions: (a) perceived capability and control (“I can do it”), and (b) perceived justification and obligation (“I have reason to do it” or “I ought to do it”). This dual-path logic aligns with contemporary motivational accounts of proactivity, which conceptualize proactive behavior as involving both goal generation and goal striving rather than being driven by resources or motivation alone (Schilpzand et al., 2018).

Building on this perspective, we argue that calling facilitates proactive work behavior among healthcare professionals by activating two complementary psychological pathways, operationalized as sense of responsibility and mission commitment. First, calling strengthens identity-based role commitment and normative attachment to one's professional role, increasing individuals' willingness to assume accountability for improving work outcomes—that is, a sense of responsibility (Pearce and Gregersen, 1991). In the proactivity domain, felt responsibility for constructive change is widely recognized as a proximal driver of change-oriented behavior, motivating individuals to exert discretionary effort to initiate improvements even in the presence of uncertainty and resistance (Morrison and Phelps, 1999). Second, calling highlights a meaning structure that emphasizes transcending self-interest and serving the public good, thereby intensifying individuals' internal commitment to occupational goals and societal contribution—that is, mission commitment (Chen et al., 2019). Mission commitment supplies a stable “reason basis” for high-cost proactive behavior, framing proactive improvement as instrumental—and sometimes necessary—for fulfilling one's professional mission, which may sustain direction and persistence under pressure and risk (Dobrow et al., 2023).

Importantly, we contend that calling will not automatically translate into proactive behavior across all contexts. For healthcare professionals, proactivity can increase accountability exposure, generate additional coordination and communication costs, and elevate perceived professional risk (Bolino et al., 2015). Whether calling-induced responsibility and mission commitment become actionable, therefore, likely depends on whether the organizational context provides normative endorsement and resource-related assurance. Leaders constitute a central source of contextual cues, and employees infer from leaders' statements and actions which behaviors are encouraged and whether reasonable risks will be supported (Kahn, 1990). From this standpoint, responsible leadership—a leadership approach emphasizing ethical conduct, fairness, stakeholder accountability, and care for employees and clients—may provide a critical foundation of psychological safety and legitimacy for proactive behavior (Siegel, 2014). When responsible leadership is high, organizations are more likely to communicate that improvement is valued, patient and employee well-being is prioritized, and reasonable trial-and-error is tolerated. Such cues should strengthen the translation of calling into both responsibility and mission commitment and increase the likelihood that these psychological states culminate in proactive work behavior. Conversely, under weak responsible leadership, calling may remain at the attitudinal level or even become a source of frustration and depletion when value-driven intentions collide with constraining realities.

Beyond serving as an applied setting, healthcare provides a theoretically informative context for examining identity-based proactivity. Healthcare work is strongly governed by professional ethics and public-service norms, which can accelerate the internalization of professional role identities and strengthen obligation-based responsibility when individuals experience a calling. At the same time, high-stakes clinical decisions and heightened accountability exposure increase the personal and interpersonal costs of proactive change-oriented action, making sustained mission commitment particularly important for maintaining engagement in improvement efforts. These contextual features also make leadership cues especially consequential, because signals about legitimacy, protection, and support shape whether professionals feel able to enact identity-based motives through proactive behavior. Accordingly, theorizing healthcare as a high-accountability, public-interest context clarifies the scope conditions under which the proposed mechanisms should be most salient.

In summary, focusing on healthcare professionals, we develop and test a theoretical model in which career calling predicts proactive work behavior through dual mediators—sense of responsibility and mission commitment—and in which responsible leadership strengthens these pathways. This study contributes to the literature in three ways. First, it extends calling research beyond the prevalent emphasis on attitudinal and well-being outcomes to proactive behavior that is central to organizational adaptability. Second, it specifies two testable psychological mechanisms that reflect a dual-path account of proactivity (“I ought to” and “I am committed to”), sharpening the explanatory precision of calling–behavior links. Third, it identifies responsible leadership as a key boundary condition for value-driven proactivity, offering actionable implications for healthcare organizations seeking to enhance resilience and service quality under resource constraints.

## 2 Theoretical background and hypotheses

### 2.1 Career calling and proactive work behavior

Career calling is typically conceptualized as a meaning-oriented and self-transcendent motivational–identity structure through which individuals construe their work as a socially valuable “mission” worthy of sustained investment, thereby pursuing purpose, significance, and contribution in their occupational lives (Xie et al., 2026). Although the notion of a “calling” historically carried religious connotations of sacred duty, contemporary organizational scholarship emphasizes its secularized meaning: calling reflects a value-based vocational identity that integrates work activities with the self-concept, moral commitment, and higher-order social purpose (King et al., 2021).

In healthcare settings, professionals operate under chronic high workload, elevated risk, and stringent procedural and regulatory constraints. Consequently, organizational adaptability and service quality hinge not only on compliant task execution but also on whether employees proactively identify problems and initiate improvements. Proactive work behavior is a prototypical change-oriented and constructive form of discretionary behavior, referring to employees' self-initiated efforts that extend beyond formal role boundaries to propose and implement improvements in work processes, performance, and organizational functioning (Wu and Parker, 2017). Because such behavior often entails additional time and effort, potential interpersonal friction, and heightened accountability exposure, it typically requires strong internal justification and role-based motivation (Peng et al., 2026).

Role identity theory suggests that individuals develop relatively stable self-definitions around the social roles they occupy and tend to enact behaviors consistent with those role meanings to maintain self-consistency and identity integrity (Ashforth and Mael, 1989). Career calling may function precisely as a motivational–identity structure that strengthens the perceived meaning of one's occupational role. When individuals experience their work as a vehicle for mission fulfillment and value realization, they are more likely to internalize the professional role as a central component of “who I am,” thereby elevating role commitment and self-regulatory standards (Chua et al., 2024). Importantly, proactive work behavior can be understood as a form of identity enactment and self-verification. Role identity theory further implies that when a professional role becomes central to the self, it generates identity standards, namely internal benchmarks of how one should think, feel, and behave as a “good” member of that profession. These standards activate a self-verification motive and create identity-consistency pressure. When individuals perceive discrepancies between their identity standards and the current state of work practices, they experience a self-regulatory impetus to reduce those discrepancies through role-consistent action, because behaving consistently with one's internalized role meanings affirms who one is and signals personal integrity.

In healthcare, where accountability exposure is salient and mistakes can carry serious consequences, proactively identifying risks, speaking up about potential problems, and initiating workflow improvements are not merely discretionary extra-role contributions. Rather, they represent a primary behavioral route through which professionals enact and validate an internalized healer-and-safeguarder identity. Accordingly, career calling should increase proactive work behavior by strengthening identity standards and motivating identity enactment, making improvement-oriented action a natural expression of internalized role meanings in high-stakes work.

When a professional role is internalized as central to one's self-schema, individuals are motivated to act in ways that confirm that identity and maintain self-consistency. For healthcare professionals with a strong calling, speaking up about risks, refining workflows, and initiating improvements are therefore not merely discretionary acts but identity-congruent behaviors that align “what I do” with “who I am” (Burmeister et al., 2025; Park et al., 2024, 2025). In turn, calling provides a compelling “reason base” for proactivity: healthcare professionals may construe identifying risks, refining workflows, and initiating improvements as morally and professionally appropriate actions that advance public welfare, rather than as optional extra-role efforts (Fehr et al., 2017).

Thus, we hypothesize:

*Hypothesis 1. Career calling is positively related to proactive work behavior among healthcare professionals*.

### 2.2 The mediating role of sense of responsibility

Sense of responsibility refers to healthcare professionals' internalized normative commitment that they ought to take ownership of work outcomes and to be accountable for initiating improvements that advance organizational functioning. It reflects a role-based sense of obligation and moral self-regulation oriented toward quality, safety, and patient well-being (Moretto et al., 2011). Importantly, this sense of responsibility is distinct from externally imposed constraints such as performance evaluation pressure or formal job requirements; rather, it stems from individuals' values and self-concept, through which organizational welfare and patient outcomes are construed as matters for which “I am personally accountable” (DeZoort and Harrison, 2018). In high-risk and highly interdependent healthcare work, such responsibility is particularly consequential. When professionals internalize quality, safety, and patient welfare as personal obligations, they are more likely—despite time pressure, process breakdowns, or resource scarcity—to proactively identify problems and drive improvements rather than merely maintaining minimal compliance or reporting issues without further action.

Career calling should strengthen this sense of responsibility. Role identity theory posits that individuals form relatively stable self-definitions around occupational roles and enact behaviors consistent with role meanings to preserve self-consistency and identity integrity (Stets and Burke, 2000). By linking daily work to higher-order social purpose (Praskova et al., 2015), calling deepens the internalization of professional role meanings and raises self-imposed moral standards. Consequently, healthcare professionals who experience stronger calling are more likely to construe “taking ownership,” “initiating improvement,” and “correcting deficiencies” as intrinsic requirements of the role rather than discretionary burdens. In this way, calling may not only elevate willingness to invest effort but also foster felt responsibility for constructive change, a proximal motivational state that compels individuals to assume accountability for improvement.

In turn, sense of responsibility should promote proactive work behavior. Proactivity typically requires employees to move beyond prescribed role boundaries to propose and implement improvements, often under uncertainty and potential personal risk (Pratama et al., 2023). Compared with general task effort, proactive behavior therefore depends more heavily on whether individuals possess a sufficiently strong internal obligation to sustain action. When healthcare professionals feel responsible for constructive improvement, they should be more likely to surface problems, offer suggestions, and follow through on implementation—thereby exhibiting higher levels of proactive work behavior.

Thus, we hypothesize:

*Hypothesis 2. Sense of responsibility mediates the positive relationship between career calling and proactive work behavior among healthcare professionals*.

### 2.3 The mediating role of mission commitment

Although conceptually related, sense of responsibility and mission commitment capture different motivational bases. Sense of responsibility is primarily obligation-based and accountability-focused, an “ought-to” orientation reflecting perceived duty to take ownership of outcomes and initiate constructive improvement. By contrast, mission commitment is purpose-based and meaning-focused, an enduring “want-to” dedication to occupational mission and public value, accompanied by sustained direction and willingness to invest. In other words, responsibility emphasizes normative obligation and moral self-regulation, whereas mission commitment emphasizes value congruence and long-term goal commitment (Batra et al., 2022; Fu et al., 2025; Li et al., 2025).

Although conceptually related, sense of responsibility and mission commitment are non-redundant and differ in motivational basis, focal target, and temporal orientation. Sense of responsibility is primarily obligation-based and accountability-focused, reflecting an “ought-to” duty to take ownership of outcomes, correct problems, and initiate constructive improvement when needed. Mission commitment is primarily purpose-based and meaning-focused, reflecting a sustained “want-to” dedication to mission goals and the public value those goals represent. Responsibility thus emphasizes accountable ownership and problem-solving demands in the near term, whereas mission commitment emphasizes enduring dedication and direction in the longer term.

In healthcare organizations, organizational mission is often tightly coupled with professional ethics and public-service mandates. Accordingly, commitment to the healthcare organization's mission can be treated as a context-specific manifestation of professionals' internalized mission in an organizational setting. This clarification aligns the conceptualization of mission commitment with the measurement approach used in this study and makes the distinct roles of responsibility and mission commitment in the model more transparent.

Mission commitment refers to health careprofessionals' internalized commitment to occupational goals and social contribution, such that they construe their work as serving an important public purpose and derive a stable legitimacy basis for sustained investment and action (Chen et al., 2019). In healthcare contexts, the occupational mandate of caring for life and serving society offers a salient and enduring value framework, making it easier for professionals to interpret day-to-day tasks as concrete enactments of helping others and contributing to the public good, thereby reinforcing mission commitment.

Career calling should provide a critical motivational–identity foundation for mission commitment. Calling emphasizes pursuing a purposeful occupational life that is socially meaningful and worthy of enduring devotion (Praskova et al., 2015). Consistent with role identity theory, when individuals internalize an occupational role as central to their self-concept, they interpret work experiences through the role's values and norms and sustain a self-narrative that is congruent with the role's meaning (Stets and Burke, 2000). Accordingly, healthcare professionals with stronger calling are more likely to incorporate “protecting health” and “caring for life” into their self-definition, experience greater alignment between daily work and personal values, and maintain a clearer sense of direction—conditions that should foster stronger mission commitment (Elangovan et al., 2010).

In turn, mission commitment should promote proactive work behavior. Proactivity requires employees to go beyond prescribed role boundaries to identify problems, voice suggestions, and implement improvements—actions that often involve additional effort and uncertain risk (Pratama et al., 2023). Mission commitment offers a robust value-based justification for such discretionary investment. When individuals are convinced that their work serves important ends, they are more willing to expend extra effort and more capable of tolerating resistance and uncertainty during change processes. Thus, healthcare professionals who experience stronger mission commitment should be more likely to view process improvement, constructive voice, and change implementation as necessary steps toward fulfilling occupational goals rather than optional extra-role choices. In this way, mission commitment constitutes a key psychological pathway through which career calling translates into proactive work behavior.

Thus, we hypothesize:

*Hypothesis 3. Mission commitment mediates the positive relationship between career calling and proactive work behavior among healthcare professionals*.

### 2.4 The moderating role of responsible leadership

Responsible leadership is theoretically central in the present context because it is particularly diagnostic for high-stakes, public-interest work. Compared with conceptually adjacent leadership constructs, responsible leadership places stronger emphasis on a stakeholder-inclusive orientation, explicit accountability for ethically justifiable actions, and dialogic engagement with employees. These features are directly relevant in healthcare, where proactive change efforts are intertwined with patient safety implications and heightened accountability exposure. In this sense, responsible leadership provides especially salient informational cues about the legitimacy of speaking up and the availability of protection and support for constructive change, thereby enabling identity-based motives to be enacted as proactive behavior.

In practice, healthcare professionals frequently juggle multiple role demands, most notably work and family responsibilities. Given the scarcity of time and energy, these competing role requirements can deplete personal resources and, in turn, reduce individuals' willingness to translate internal motives into additional effort and discretionary, beyond-role behavior (Trougakos et al., 2015). Thus, even when healthcare professionals experience a strong career calling, real-world constraints may prevent this motivation from crystallizing into a stable sense of responsibility and sustained improvement-oriented action. Under such conditions, whether the organization—particularly its agents—provides support becomes a key boundary condition that determines whether calling “takes hold” and becomes behaviorally consequential. In the present study, we focus on responsible leadership specifically as stakeholder-oriented ethical prioritization (e.g., patient safety and staff well-being), leader accountability and fair treatment that legitimizes constructive voice, and resource/psychological-safety provision that reduces the perceived risks of initiating change. These facets are particularly salient in healthcare, where proactive improvement often involves high accountability exposure and uncertainty (Dong and Zhong, 2021; Kepplinger et al., 2024; Özkan et al., 2023).

Social information processing theory posits that employees extract and interpret social cues from their work environment to infer which behaviors are expected, valued, and supported, and then calibrate their understandings of role obligations and behavioral consequences accordingly (Salancik and Pfeffer, 1978). Responsible leadership, characterized by signaling concern for employee well-being, emphasizing ethical and fair treatment, and demonstrating willingness to assume accountability for reasonable actions, provides salient cues that proactive ownership and constructive responsibility are legitimate and supported (Brown et al., 2005). When such cues are strong, employees are more likely to interpret the context as one in which constructive “taking responsibility” is encouraged and the risks of acting on one's values are more likely to be buffered. Consequently, healthcare professionals should be more inclined to internalize and strengthen role-based responsibility rather than treat responsibility as an aspirational ideal that is difficult to enact.

In addition to offering normative cues, responsible leadership may also operate through a resource-based mechanism. By providing instrumental and socioemotional support, responsible leaders can mitigate resource loss associated with work–family tensions and reduce the psychological and practical costs of “stepping up” (Hu et al., 2021). Such support should enhance perceived psychological safety and controllability, allowing employees to sustain the normative belief that “I ought to take ownership” as a stable sense of responsibility rather than having it eroded by chronic strain and uncertainty.

Taken together, responsible leadership should amplify the positive relationship between career calling and sense of responsibility. In contexts characterized by higher responsible leadership, healthcare professionals' calling is more likely to translate into an internal commitment to constructive improvement and role accountability. Conversely, when responsible leadership is weak, calling-induced responsibility tendencies may be less likely to consolidate because the perceived support and safety boundaries necessary for sustained ownership are absent.

*Hypothesis 4. Responsible leadership positively moderates the relationship between career calling and healthcare professionals' sense of responsibility, such that the relationship is stronger when responsible leadership is high rather than low*.

Social information processing theory suggests that employees extract and interpret social cues from the organizational environment to construct cognitive frameworks regarding work meaning, organizational expectations, and their role positioning, and they subsequently adjust attitudes and behavior accordingly (Salancik and Pfeffer, 1978). Responsible leadership—characterized by ethical concern, fair treatment, and accountability to employees as well as broader stakeholders—can provide recurring signals that the organization values human well-being and public purpose and supports legitimate improvement and responsible ownership (Nakra and Kashyap, 2024). These signals may facilitate the translation of career calling into mission commitment in at least two ways. First, they offer social validation for meaning interpretation, making it easier for healthcare professionals to construe demanding daily tasks as integral to serving the public good and fulfilling an occupational mission. Second, responsible leadership can coordinate resources and establish psychological safety boundaries, thereby reducing the cognitive and emotional costs of meaning making under high pressure and uncertainty. Together, these cues and supports should strengthen mission commitment—defined as an internalized commitment to occupational goals and social contribution, accompanied by a sustained sense of direction.

Accordingly, when responsible leadership is high, healthcare professionals are more likely to receive consistent, supportive cues from leaders' words and enacted practices, which should reinforce their understanding of and identification with the value-laden goals implied by calling and thereby heighten mission commitment. When responsible leadership is low, however, contextual cues may be insufficient to sustain meaning construction, and the positive effect of career calling on mission commitment is less likely to fully materialize.

*Hypothesis 5. Responsible leadership positively moderates the relationship between career calling and healthcare professionals' mission commitment, such that the relationship is stronger when responsible leadership is high rather than low*.

### 2.5 Conditional indirect effects: moderated mediation by responsible leadership

In healthcare contexts characterized by high risk, high intensity, and competing role demands, engaging in proactive work behavior often entails substantial resource burdens and psychological costs. Social information processing theory suggests that employees extract and interpret social cues from their organizational environment to judge which behaviors are encouraged, whether risks are manageable, and whether discretionary investment is worthwhile, and they calibrate their attitudes and behaviors accordingly (Salancik and Pfeffer, 1978). Responsible leadership, which emphasizes ethical care, fair treatment, and accountability to multiple stakeholders, can provide clear and credible support signals through leaders' consistent words, deeds, and resource-coordination actions. Such signals communicate that the organization values employee well-being, legitimizes constructive ownership and improvement, and is willing to provide protective boundaries for reasonable attempts (Hu et al., 2021). These contextual cues may alleviate resource strain stemming from work–family conflict and emotional labor demands while enhancing employees' perceived psychological safety and legitimacy. As a result, career calling should be more effectively translated into stronger sense of responsibility and mission commitment, which in turn increase the likelihood of proactive work behavior.

Accordingly, when responsible leadership is high, the value commitment and role identity implied by career calling should be more likely to translate into proactive work behavior through two complementary psychological pathways: sense of responsibility (“I ought to initiate constructive change”) and mission commitment (“my work advances important goals and public value”). When responsible leadership is low, however, supportive cues are weaker and the positive inclinations embedded in calling are less likely to consolidate into these proximal motivational states, thereby attenuating the indirect effect of calling on proactive work behavior.

*Hypothesis 6. Responsible leadership moderates the indirect effect of career calling on proactive work behavior via sense of responsibility, such that the indirect effect is stronger when responsible leadership is high rather than low*.
*Hypothesis 7. Responsible leadership moderates the indirect effect of career calling on proactive work behavior via mission commitment, such that the indirect effect is stronger when responsible leadership is high rather than low*.

**Figure 1:**
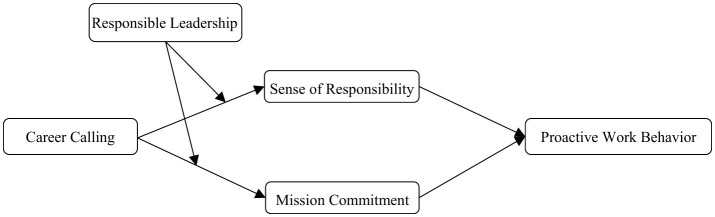
Research model.

In summary, the theoretical concept is shown in Figure 1.

## 3 Method

### 3.1 Sample and procedure

This study surveyed healthcare professionals from representative medical institutions in Eastern, Central, and North China. Data were collected using on-site questionnaires distributed and retrieved in multiple waves. Specifically, participants were recruited from several tertiary hospitals (Grade A, Class III) and community healthcare institutions located in Jiangsu, Hubei, Beijing, Shanghai, and Guangdong. With the assistance of human resources departments, questionnaires were distributed to eligible staff members. Participants were informed that all responses would be collected anonymously and kept strictly confidential, and that the data would be used solely for academic research purposes.

To reduce the risk of common method bias, a three-wave, time-lagged research design was employed. Sampling followed a multi-site, purposive approach combined with on-site convenience recruitment. Specifically, we intentionally selected tertiary hospitals (Grade A, Class III) and community healthcare institutions across Eastern, Central, and North China to capture variability in organizational context and clinical work demands, and then invited eligible staff members within each institution to participate. Eligibility criteria included being a frontline healthcare professional (e.g., physician, nurse, allied health staff), working full-time, and having at least 6 months of tenure to ensure adequate exposure to local leadership and work routines. To match responses across waves while preserving anonymity, participants created a self-generated identification code (e.g., based on personal cues known only to them), which was used solely for survey matching and was not linked to any identifiable information. Participation was voluntary, and respondents were reminded that they could withdraw at any time without penalty. Data collection was conducted over a period of more than 3 months, from June to September 2025, with a 1-month interval between consecutive waves. At Time 1, career calling and responsible leadership, as well as demographic variables, were assessed. At Time 2, sense of responsibility and mission commitment. were assessed. At Time 3, proactive work behavior was measured. In total, 550 questionnaires were distributed, and 417 valid responses were obtained, yielding an effective response rate of 75.81%.

Sample characteristics were as follows. Gender: 59.47% male, 40.53% female. Age: 18–25 years 23.98%, 26–35 years 45.32%, 36–55 years 23.74%, 56 years and older 6.95%. Educational attainment: junior college or below 14.15%, bachelor's degree 50.60%, master's degree 26.14%, doctorate or above 9.11%. Organizational tenure: 3 years or less 16.79%, 3–5 years 41.25%, 6–9 years 27.10%, 10 years or more 14.87%.

### 3.2 Measures

The survey was conducted in Chinese; we translated the original items of each measure into Chinese using Brislin (1970) forward and backward process. Unless otherwise stated, items were evaluated on a five-point Likert scale that ranged from 1 (*strongly disagree*) to 5 (*strongly agree*).

***Career calling***. Career calling was measured using the 12-item unidimensional scale developed by Dobrow and Tosti-Kharas (2011). A sample item is: “I enjoy doing my work more than anything else”. (Cronbach's α = 0.961).

***Sense of responsibility*. **Sense of responsibility was measured using the seven-item felt obligation scale developed by Eisenberger et al. (2001), which captures employees' perceived obligation to care about the organization and help it reach its goals. A sample item is, “I feel a responsibility to do my best to help the organization achieve its objectives.” (Cronbach's α = 0.937).

***Mission commitment*. **Mission commitment was assessed as commitment to the healthcare organization's mission, which we conceptualize as a context-specific manifestation of professionals' internalized mission given the tight coupling of organizational mission with professional ethics in healthcare. Mission commitment was assessed using five items developed by Brown and Yoshioka (2003) to capture employees' commitment to and identification with the organization's mission. A sample item is, “I am well aware of the direction and mission of my organization.” (Cronbach's α = 0.933).

***Proactive work behavior***. Proactive work behavior was measured using the seven-item scale developed by Frese et al. (1997). A sample item is, “Whenever something goes wrong, I search for a solution immediately.” The scale demonstrated high internal consistency (Cronbach's α = 0.960).

***Responsible leadership*. **Responsible leadership was assessed by employees using five items developed by Voegtlin (2011). A sample item was “My immediate supervisor demonstrates awareness of the relevant stakeholder” (α = 0.957).

***Control variables*. **Established studies have found that gender, age, education, and organizational tenure affect the cognitive evaluation of variables (Dobrow and Tosti-Kharas, 2011; Elangovan et al., 2010; Hu et al., 2021; Xie et al., 2026; Waqas et al., 2021; Zhang et al., 2023; Lu et al., 2024), and thus this study controls for the above variables.

## 4 Results

### 4.1 Common method bias, discriminant validity, and convergent validity

Since the data were all self-assessed by the employees, in order to avoid the effect of common method bias (Podsakoff et al., 2003), the Harman single-factor test found that the unrotated first factor explained 37.66% of the variation, less than 40% (Zhou and George, 2001). Further analysis using the common latent factor method by adding the common method bias factor together with the original five factors showed no significant improvement in the model fit index (Δχ^2^/*df* = 0.057, ΔCFI = 0.007, ΔTFI = 0.002, ΔRMSEA = 0.001, ΔRMSEA = 0.005). Therefore, the problem of common method bias is not serious.

In this study, Mplus 8.3 software was used to determine the degree of compliance of the scale question items with the measurement objectives by confirmatory factor analysis. In general, the model is considered to fit well if the χ^2^/*df* is less than 5 (the smaller, the better), the RMSEA is less than 0.1 (the smaller, the better), the SRME is less than 0.05 (the smaller, the better), the CFI and TLI are all above 0.8 (the larger, the better). According to Table 1, it was found that the five-factor model fit better (χ^2^/*df* = 3.329, CFI = 0.912, TLI = 0.905, RMSEA = 0.075, SRMR = 0.045) than the others, which indicates that the discriminant validity between the core variables in this study was significant.

**Table 1:** Results of confirmatory factor analysis.

Models	χ^2^	*df*	χ^2^*/df*	CFI	TLI	RMSEA	SRMR
Five-factor model^a^	1,943.990	584	3.329	0.912	0.905	0.075	0.045
Four-factor model^b^	3,860.862	588	6.566	0.787	0.772	0.116	0.124
Three-factor model^c^	5,351.405	591	9.055	0.690	0.670	0.139	0.143
Two-factor model^d^	7,726.756	593	13.030	0.536	0.507	0.170	0.146
One-factor model^e^	9,451.756	594	15.912	0.424	0.389	0.189	0.155

^a^Each variable was a factor alone.

^b^Career calling and sense of responsibility was combined into one factor.

^c^Career calling, sense of responsibility, and mission commitment were combined into one factor.

^d^Career calling, sense of responsibility, mission commitment, and proactive work behavior were combined into one factor.

^e^Combine all variables.

### 4.2 Descriptive statistics

Descriptive statistics and Pearson correlation coefficients are presented in Table 2. Career calling was significantly and positively correlated with sense of responsibility (*r* = 0.428, *p* < 0.001), mission commitment (*r* = 0.343, *p* < 0.001), and proactive work behavior (*r* = 0.422, *p* < 0.001). Additionally, sense of responsibility was positively associated with proactive work behavior (*r* = 0.535, *p* < 0.001), and mission commitment was positively associated with proactive work behavior (*r* = 0.449, *p* < 0.001). These results provide preliminary support for the hypothesized relationships.

**Table 2:** Descriptive statistics and correlation analysis.

Variables	*M*	*SD*	1	2	3	4	5	6	7	8	9
1. Gender	1.405	0.492	1								
2. Age	2.137	0.859	−0.063	1							
3. Education	2.302	0.823	−0.072	0.040	1						
4. Organizational tenure	2.400	0.936	−0.082	0.356^***^	0.024	1					
5. Career calling	3.417	0.608	−0.041	0.005	0.066	0.042	1				
6. Sense of responsibility	4.243	0.591	−0.102^*^	0.104^*^	−0.022	0.057	0.428^***^	1			
7. Mission commitment	3.819	0.761	−0.028	0.053	−0.024	0.117^*^	0.343^***^	0.504^***^	1		
8. Proactive work behavior	4.039	0.677	−0.126^*^	0.094	0.072	0.120^*^	0.422^***^	0.535^***^	0.449^***^	1	
9. Responsible leadership	3.846	0.833	−0.083	0.077	0.019	0.087	0.433^***^	0.333^***^	0.339^***^	0.451^***^	1

*N* = 417.

^*^p < 0.05,

^***^p < 0.001 (two-tail test).

### 4.3 Hypotheses testing

We used the Mplus 8.3 path analysis method to test the hypotheses (Hayes, 2013), and the specific results are shown in Table 3. Our results revealed that career calling was positively associated with proactive work behavior (β = 0.183, *p* < 0.001). Moreover, career calling was positively associated with both sense of responsibility (β = 0.370, *p* < 0.001) and mission commitment (β = 0.337, *p* < 0.01). Sense of responsibility was positively associated with proactive work behavior (β = 0.336, *p* < 0.001), whereas mission commitment was positively associated with proactive work behavior (β = 0.132, *p* < 0.05). As such, career calling had a significant indirect effect on proactive work behavior via sense of responsibility (*indirect effect* = 0.124, *95% CI* [0.077, 0.184]), as well as a significant indirect effect on proactive work behavior via mission commitment (*indirect effect* = 0.044, *95% CI* [0.011, 0.098]). Thus, Hypotheses 1, 2, and 3 were supported.

**Table 3:** Results of path analysis.

Variables	Sense of responsibility		Mission commitment		Proactive work behavior	
	β	*SE*	β	*SE*	β	*SE*
Control variables						
Gender	−0.076	0.051	0.020	0.071	−0.066	0.053
Age	0.062^*^	0.028	0.006	0.038	0.011	0.036
Education	−0.036	0.030	−0.038	0.042	0.057	0.033
Organizational tenure	−0.013	0.030	0.061	0.038	0.026	0.028
Independent variable						
Career calling	0.370^***^	0.048	0.337^***^	0.074	0.183^**^	0.053
Mediating variables						
Sense of responsibility					0.336^***^	0.056
Mission commitment					0.132^*^	0.052
Moderating variable						
Responsible leadership	0.169^***^	0.041	0.266^***^	0.055	0.249^***^	0.060
Interaction						
Career calling × responsible leadership	0.141^**^	0.047	0.168^*^	0.069	0.173^*^	0.071
*R* ^**2**^	25%		19%		42%	

*N* = 417.

^*^p < 0.05,

^**^p < 0.01,

^***^p < 0.001 (two-tail test).

**Figure 2:**
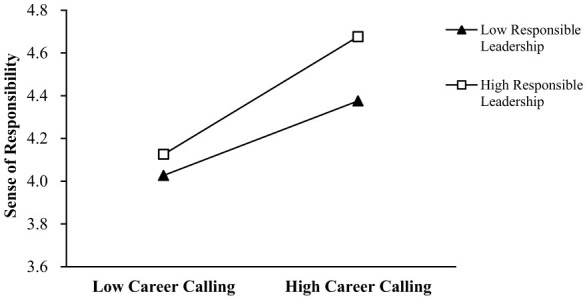
The moderating effect of responsible leadership on the relationship between career calling and sense of responsibility.

**Figure 3:**
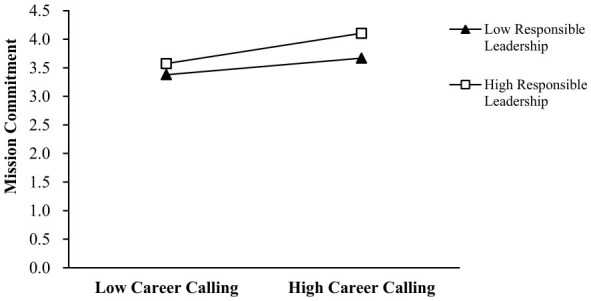
The moderating effect of responsible leadership on the relationship between career calling and felt obligation.

Our results revealed that the interaction term between career calling and responsible leadership significantly affected sense of responsibility (β = 0.141, *p* < 0.001) and mission commitment (β = 0.168, *p* < 0.05). We drew simple slope tests and plotted interactions in Figures 2, 3. Thus, Hypotheses 4 and 5 were supported.

Next, we assessed the hypothesized moderated indirect effects. As reported in Table 4, career calling had significant indirect effect on proactive work behavior via sense of responsibility at higher levels of responsible leadership (*indirect effect* = 0.164, *95% CI* [0.101, 0.246]) than at lower levels (*indirect effect* = 0.085, *95% CI* [0.043, 0.145]), with significant differences (*index* = 0.079, *95% CI* [0.024, 0.148]). Similarly, career calling had significant indirect effect on proactive work behavior via mission commitment at higher levels of responsible leadership (*indirect effect* = 0.063, *95% CI* [0.016, 0.137]) than at lower levels (*indirect effect* = 0.026, *95% CI* [0.003, 0.073]), with significant differences (*index* = 0.037, *95% CI* [0.006, 0.102]). Thus, Hypotheses 6 and 7 were supported.

**Table 4:** Results of moderated indirect effects.

Mediators	Moderator	Effect	Boot SE	Boot LLCI	Boot ULCI
Sense of responsibility	M – 1SD	0.164	0.037	0.101	0.246
	M + 1SD	0.085	0.026	0.043	0.145
	Index	0.079	0.031	0.024	0.148
Mission commitment	M – 1SD	0.063	0.030	0.016	0.137
	M + 1SD	0.026	0.017	0.003	0.073
	Index	0.037	0.023	0.006	0.102

## 5 Discussion

Our findings also speak directly to an ongoing debate in the calling and proactivity literatures about whether meaning-laden orientations reliably translate into discretionary, change-oriented action. Much of the calling research has emphasized attitudinal, well-being, and performance outcomes, while providing comparatively less evidence on behaviors that are effortful, uncertain, and potentially costly, which are defining features of proactivity. By showing that career calling predicts proactive work behavior through two proximal motivational states, our study extends prior accounts that often treat the calling-to-outcome process as a relatively unitary pathway and suggests a more differentiated translation mechanism. Specifically, the coexistence of an obligation-based route through sense of responsibility and a purpose-based route through mission commitment helps address implicit disagreements about whether calling operates primarily via duty-like obligation or meaning-based dedication, indicating that both logics can jointly and uniquely energize proactive action. Moreover, consistent with social information processing perspectives, the moderating role of responsible leadership underscores that values do not automatically become action; employees appear to rely on leadership cues to infer whether taking initiative is legitimate and sufficiently safe. This may be especially consequential in high-accountability healthcare settings, where proactive change can entail heightened interpersonal and accountability risks. Our theorizing suggests that the proposed mechanisms may be especially salient in work systems characterized by high professionalization, strong ethical mandates, and heightened accountability exposure, such as healthcare. In contexts where accountability is weaker or professional norms are less tightly coupled with organizational missions, career calling may be less likely to translate into responsibility-based ownership or may rely on different contextual cues. Future research could examine whether the relative importance of responsibility, mission commitment, and leadership cues varies across occupations with different levels of ethical mandate and accountability exposure.

### 5.1 Theoretical implications

This study strengthens theorizing on value-driven proactivity by specifying how and when career calling becomes behaviorally consequential. First, by linking calling to proactive work behavior in healthcare, we extend the calling literature beyond its predominant focus on attitudinal and well-being outcomes to a discretionary, change-oriented criterion that is often effortful and risky. Importantly, this finding is theoretically consistent with role identity accounts: calling can heighten identity centrality and self-regulatory standards, making constructive initiative a form of identity enactment rather than merely an extra-role choice.

Second, our results refine the “value-to-action” mechanism by distinguishing two proximal motivational states that carry calling's influence. Sense of responsibility captures an obligation-based, accountability-focused motive that can mobilize ownership and corrective change under moral and professional norms, whereas mission commitment captures a purpose-based dedication that sustains discretionary investment through meaning and goal commitment. Demonstrating incremental indirect effects through both routes advances conceptual precision and suggests that calling may energize proactivity through multiple motivational bases rather than a single, undifferentiated pathway.

Third, by showing that responsible leadership strengthens the first-stage links from calling to both mediators, our study integrates role identity and social information processing perspectives. The pattern indicates that identity-laden values do not automatically translate into action; employees rely on leadership-provided cues to infer whether constructive change is legitimate and sufficiently safe. Responsible leadership therefore functions as an interpretive and enabling context that helps calling consolidate into stable motivational states and, in turn, facilitates enacted proactivity. Taken together, the findings offer a more systemic account of value-driven proactivity that connects internalized role meaning with contextual cue interpretation and boundary conditions.

### 5.2 Practical implications

Our findings suggest that fostering proactive improvement in healthcare requires aligning individual motivation with an enabling organizational context. Priority should be given to organizational levers, because responsible leadership emerged as a key boundary condition that helps transform calling into responsibility and mission commitment and, in turn, proactive behavior. Healthcare organizations can therefore invest in leadership development that emphasizes stakeholder-oriented ethical priorities (e.g., patient safety and staff well-being), fair and accountable treatment, and the provision of psychological safety and resources that legitimize constructive voice and trial-and-error. Practically, leaders should make improvement expectations explicit, invite input, respond non-defensively to suggestions, and allocate time and resources for frontline-driven quality improvement.

Second, organizations can strengthen individual-level motivational foundations by supporting career calling through recruitment, selection, and socialization practices that emphasize mission alignment and professional identity internalization (Sarfraz et al., 2022). For example, realistic job previews, onboarding that clarifies the organization's mission and public value, and career development practices that connect daily tasks to patient-centered outcomes may help employees interpret their work as meaningful and sustain mission commitment.

Finally, because proactive behavior can be effortful, organizations should pair these initiatives with workload-sensitive systems (e.g., staffing adequacy, protected improvement time, and recovery opportunities) to prevent role overload and sustain proactivity over time.

### 5.3 Limitations and future research

First, the study's design and measurement strategy may raise concerns about common method bias. Core variables were primarily obtained from the same respondents' self-reports. Although the use of a time-lagged design helps reduce same-source bias, it cannot fully rule out inflation due to social desirability or consistency motives. Future research could adopt multi-source and multi-method approaches. for example, incorporating supervisor or coworker ratings of proactive work behavior and objective organizational indicators (e.g., participation in quality-improvement initiatives, implemented suggestions, or contributions to process-optimization projects). Longitudinal designs or experience sampling methods (ESM) would also be valuable for capturing within-person dynamics and strengthening robustness and ecological validity. Relatedly, proactive work behavior may entail non-trivial costs, particularly in resource-constrained healthcare settings. Although proactivity can improve quality and resilience, it may also increase role overload, accountability exposure, and the risk of citizenship fatigue—especially when responsible leadership is weak and employees perceive limited psychological safety or support for trial-and-error. Future research could examine potential “too-much-of-a-good-thing” dynamics, such as whether calling-driven proactivity becomes unsustainable under chronic strain, and identify buffering conditions (e.g., staffing adequacy, recovery experiences, or team-level support) that help maintain proactive efforts without depletion.

Second, the cultural and institutional context of the sample not only bounds generalizability but also offers a theoretically informative lens for interpreting the calling proactivity process. This study was conducted in Chinese healthcare organizations. In contexts characterized by collectivistic orientations and strong professional-ethical norms, career calling may be more readily construed as social responsibility and collective commitment, thereby more directly fostering mission commitment, sense of responsibility, and proactivity. From a role identity perspective, such norms may intensify the internalization of professional role meanings and heighten self-regulatory standards, making “taking charge” more identity-congruent. From a social information processing perspective, shared ethical expectations and collectivistic norms may also amplify the salience and credibility of leadership cues that legitimize constructive change, thereby strengthening the first-stage paths in our model. In cultures that emphasize individual choice, self-actualization, and occupational mobility, however, the meaning of calling and its behavioral consequences may follow different patterns. Future work should conduct cross-cultural comparisons or multi-country studies to test how cultural values and institutional differences shape the proposed mechanisms and boundary conditions and to clarify the scope conditions of the present findings.

Third, there remains scope to expand the set of mechanisms and boundary conditions. We focused on two central psychological pathways, mission commitment and sense of responsibility, and examined responsible leadership as a key contextual moderator. Yet calling may also promote proactivity through additional routes, such as self-efficacy and perceived control, career-growth expectations, moral emotions, or psychological capital. Moreover, beyond responsible leadership, other leadership styles (e.g., empowering, transformational, and ethical leadership) and more specific forms of support (e.g., family-supportive supervision and organizational support practices) may represent boundary conditions at different levels. Future research could extend the model to incorporate multiple pathways while maintaining theoretical parsimony, and examine the relative and interactive effects of leadership and institutional supports in translating values into action thereby developing a more comprehensive account of how calling becomes behaviorally consequential.

## 6 Conclusion

This study examined how career calling translates into proactive work behavior among healthcare professionals. By identifying dual mediating mechanisms (sense of responsibility and mission commitment) and clarifying responsible leadership as a key boundary condition, the findings enrich role identity and social information processing perspectives on value-driven proactivity. We hope this work encourages future research to test these mechanisms across contexts and over time.

## Data Availability

The raw data supporting the conclusions of this article will be made available by the authors, without undue reservation.

## References

[B1] Ashforth B. E. Mael F. (1989). Social identity theory and the organization. Acad. Manag. Rev. 14, 20–39. doi: 10.2307/258189

[B2] Batra S. Orban J. Zhang H. Guterbock T. M. Butler L. A. Bogucki C. . (2022). Analysis of social mission commitment at dental, medical, and nursing schools in the US. JAMA Netw. Open 5:e2210900. doi: 10.1001/jamanetworkopen.2022.10900 35532935 PMC9086841

[B3] Bolino M. C. Hsiung H.-H. Harvey J. LePine J. A. (2015). “Well, I'm tired of tryin'!” organizational citizenship behavior and citizenship fatigue. J. Appl. Psychol. 100, 56–74. doi: 10.1037/a0037583 25111252

[B4] Brislin R. W. (1970). Back-translation for cross-cultural research. J. Cross Cult. Psychol. 1:185. doi: 10.1177/135910457000100301

[B5] Brown M. E. Treviño L. K. Harrison D. A. (2005). Ethical leadership: a social learning perspective for construct development and testing. Organ. Behav. Hum. Decis. Process. 97, 117–134. doi: 10.1016/j.obhdp.2005.03.002

[B6] Brown W. A. Yoshioka C. F. (2003). Mission attachment and satisfaction as factors in employee retention. Nonprofit Manag. Lead. 14, 5–18. doi: 10.1002/nml.18

[B7] Burmeister A. Song Y. Wang M. Hirschi A. (2025). Understanding knowledge sharing from an identity-based motivational perspective. J. Manag. 51, 2946–2979. doi: 10.1177/01492063241248106

[B8] Chen Y. Kim E. S. Koh H. K. Frazier A. L. VanderWeele T. J. (2019). Sense of mission and subsequent health and well-being among young adults: an outcome-wide analysis. Am. J. Epidemiol. 188, 664–673. doi: 10.1093/aje/kwz009 30649174 PMC6438813

[B9] Chua N. Miska C. Mair J. Stahl G. K. (2024). Purpose in management research: navigating a complex and fragmented area of study. Acad. Manag. Ann. 18, 755–787. doi: 10.5465/annals.2022.0186

[B10] Cutler D. M. Ghosh K. Messer K. L. Raghunathan T. Rosen A. B. Stewart S. T. (2022). A satellite account for health in the United States. Am. Econ. Rev. 112, 494–533. doi: 10.1257/aer.20201480 35529584 PMC9070842

[B11] Demerouti E. Bakker A. B. Nachreiner F. Schaufeli W. B. (2001). The job demands-resources model of burnout. J. Appl. Psychol. 86, 499–512. doi: 10.1037/0021-9010.86.3.499 11419809

[B12] DeZoort F. T. Harrison P. D. (2018). Understanding auditors' sense of responsibility for detecting fraud within organizations. J. Bus. Ethics 149, 857–874. doi: 10.1007/s10551-016-3064-3

[B13] Dobrow S. R. Tosti-Kharas J. (2011). Calling: the development of a scale measure. Pers. Psychol. 64, 1001–1049. doi: 10.1111/j.1744-6570.2011.01234.x

[B14] Dobrow S. R. Weisman H. Heller D. Tosti-Kharas J. (2023). Calling and the good life: a meta-analysis and theoretical extension. Adm. Sci. Q. 68, 508–550. doi: 10.1177/00018392231159641

[B15] Dong W. Zhong L. (2021). Responsible leadership fuels innovative behavior: the mediating roles of socially responsible human resource management and organizational pride. Front. Psychol. 12:787833. doi: 10.3389/fpsyg.2021.787833 34956013 PMC8703137

[B16] Eisenberger R. Armeli S. Rexwinkel B. Lynch P. D. Rhoades L. (2001). Reciprocation of perceived organizational support. J. Appl. Psychol. 86, 42–51. doi: 10.1037/0021-9010.86.1.42 11302232

[B17] Eisenberger R. Huntington R. Hutchison S. Sowa D. (1986). Perceived organizational support. J. Appl. Psychol. 71, 500–507. doi: 10.1037/0021-9010.71.3.500

[B18] Elangovan A. R. Pinder C. C. McLean M. (2010). Callings and organizational behavior. J. Vocat. Behav. 76, 428–440. doi: 10.1016/j.jvb.2009.10.009

[B19] Fehr R. Fulmer A. Awtrey E. Miller J. A. (2017). The grateful workplace: a multilevel model of gratitude in organizations. Acad. Manag. Rev. 42, 361–381. doi: 10.5465/amr.2014.0374

[B20] Frese M. Fay D. Hilburger T. Leng K. Tag A. (1997). The concept of personal initiative: operationalization, reliability and validity in two German samples. J. Occup. Organ. Psychol. 70, 139–161. doi: 10.1111/j.2044-8325.1997.tb00639.x

[B21] Fu H. Hu H. Wang Z. (2025). Power hierarchy and followership behavior: felt responsibility for constructive change as a mediator and status hierarchy as a moderator. Balt. J. Manag. 20, 544–559. doi: 10.1108/BJM-11-2024-0752

[B22] Gaines J. Jermier J. M. (1983). Emotional exhaustion in a high stress organization. Acad. Manag. J. 26, 567–586. doi: 10.2307/255907

[B23] Griffin M. A. Grote G. (2020). When is more uncertainty better? A model of uncertainty regulation and effectiveness. Acad. Manag. Rev. 45, 745–765. doi: 10.5465/amr.2018.0271

[B24] Hayes A. F. (2013). Introduction to Mediation, Moderation, and Conditional Process Analysis: A Regression-Based Approach. New York, NY: Guilford Press.

[B25] Hu W. Zhao F. Zhang Y. Lu T. (2021). Green human resource practice and team innovation performance: roles of team boundary-spanning behavior and responsible leadership. Transform. Bus. Econ. 20, 1745–1765.

[B26] Kahn W. A. (1990). Psychological conditions of personal engagement and disengagement at work. Acad. Manag. J. 33, 692–724. doi: 10.5465/256287

[B27] Kajaria-Montag H. Freeman M. Scholtes S. (2024). Continuity of care increases physician productivity in primary care. Manage. Sci. 70, 7943–7960. doi: 10.1287/mnsc.2021.02015

[B28] Kepplinger A. Braun A. Fringer A. Roes M. (2024). Opportunities for nurses to address employee voice in health care providers: a scoping review. BMC Nurs. 23:651. doi: 10.1186/s12912-024-02331-y 39272093 PMC11401326

[B29] King C. Madera J. M. Lee L. Murillo E. Baum T. Solnet D. (2021). Reimagining attraction and retention of hospitality management talent–a multilevel identity perspective. J. Bus. Res. 136, 251–262. doi: 10.1016/j.jbusres.2021.07.044

[B30] Lai A. Y. Wee K. Z. Frimpong J. A. (2024). Proactive behaviors and health care workers: a systematic review. Health Care Manage. Rev. 49, 239–251. doi: 10.1097/HMR.0000000000000409 38757911

[B31] Li X. Zhao L. Hai S. (2025). CEO's entrepreneurial orientation and employees' innovative behaviour: the roles of environmental turbulence and felt responsibility. Humanities Soc. Sci. Commun. 12:136. doi: 10.1057/s41599-025-04500-z

[B32] Lu S. Sun Z. Huang M. (2024). The impact of digital literacy on farmers' pro-environmental behavior: an analysis with the theory of planned behavior. Front. Sustain. Food Syst. 8:1432184. doi: 10.3389/fsufs.2024.1432184

[B33] Moretto G. Walsh E. Haggard P. (2011). Experience of agency and sense of responsibility. Conscious. Cogn. 20, 1847–1854. doi: 10.1016/j.concog.2011.08.014 21920776

[B34] Morrison E. W. Phelps C. C. (1999). Taking charge at work: extrarole efforts to initiate workplace change. Acad. Manag. J. 42, 403–419. doi: 10.2307/257011

[B35] Nakra N. Kashyap V. (2024). Responsible leadership and organizational sustainability performance: investigating the mediating role of sustainable HRM. Int. J. Prod. Perform. Manag. 74, 409–426. doi: 10.1108/IJPPM-03-2023-0115

[B36] Özkan O. S. Huertas-Valdivia I. Üzüm B. (2023). Fostering employee promotive voice in hospitality: the impact of responsible leadership. Tour. Manag. Perspect. 49:101186. doi: 10.1016/j.tmp.2023.101186

[B37] Park I. J. Shi X. (C.), Kim, P. B. Park J. (2024). The progressive impact of career calling on voice behaviors through learning goal orientation: a moderated mediation model with affect spin. Int. J. Hosp. Manag. 123:103893. doi: 10.1016/j.ijhm.2024.103893

[B38] Park M. Chae H. Tian X. (2025). Proactive but not always creative: a moderated mediation model of creative identity and psychological safety. Acta Psychol. 260:105667. doi: 10.1016/j.actpsy.2025.105667 41038015

[B39] Parker S. K. Williams H. M. Turner N. (2006). Modeling the antecedents of proactive behavior at work. J. Appl. Psychol. 91, 636–652. doi: 10.1037/0021-9010.91.3.636 16737360

[B40] Pearce J. L. Gregersen H. B. (1991). Task interdependence and extrarole behavior: a test of the mediating effects of felt responsibility. J. Appl. Psychol. 76, 838–844. doi: 10.1037/0021-9010.76.6.838

[B41] Peng J. Nie Q. Chen X. (2026). Interpreting and reacting to followers' proactive work behaviors: the role of leaders' proactive implicit followership theories. J. Bus. Res. 205:115904. doi: 10.1016/j.jbusres.2025.115904

[B42] Podsakoff P. M. Mackenzie S. B. Lee J.-Y. Podsakoff N. P. (2003). Common method biases in behavioral research: a critical review of the literature and recommended remedies. J. Appl. Psychol. 88, 879–903. doi: 10.1037/0021-9010.88.5.879 14516251

[B43] Praskova A. Creed P. A. Hood M. (2015). The development and initial validation of a career calling scale for emerging adults. J. Career Assess. 23, 91–106. doi: 10.1177/1069072714523089

[B44] Pratama A. S. Sridadi A. R. Eliyana A. Anggraini R. D. Kamil N. L. M. (2023). A systematic review of proactive work behavior: future research recommendation. J. Behav. Sci. 18, 136–151.

[B45] Rindova V. Courtney H. (2020). To shape or adapt: knowledge problems, epistemologies, and strategic postures under knightian uncertainty. Acad. Manag. Rev. 45, 787–807. doi: 10.5465/amr.2018.0291

[B46] Salancik G. R. Pfeffer J. (1978). A social information processing approach to job attitudes and task design. Adm. Sci. Q. 23, 224–231. doi: 10.2307/2392563 10307892

[B47] Sarfraz M. Hafeez H. Abdullah M. I. Ivascu L. Ozturk I. (2022). The effects of the COVID-19 pandemic on healthcare workers' psychological and mental health: The moderating role of felt obligation. Work 71, 539–550. doi: 10.3233/WOR-211073 35253715

[B48] Schabram K. Nielsen J. Thompson J. (2023). The dynamics of work orientations: an updated typology and agenda for the study of jobs, careers, and callings. Acad. Manag. Ann. 17, 405–438. doi: 10.5465/annals.2021.0153

[B49] Schilpzand P. Houston L. Cho J. (2018). Not too tired to be proactive: daily empowering leadership spurs next-morning employee proactivity as moderated by nightly sleep quality. Acad. Manag. J. 61, 2367–2387. doi: 10.5465/amj.2016.0936

[B50] Siegel D. S. (2014). Responsible leadership. Acad. Manag. Perspect. 28, 221–223. doi: 10.5465/amp.2014.0081

[B51] Stets J. E. Burke P. J. (2000). Identity theory and social identity theory. Soc. Psychol. Q. 63, 224–237. doi: 10.2307/2695870

[B52] Trougakos J. P. Beal D. J. Cheng B. H. Hideg I. Zweig D. (2015). Too drained to help: a resource depletion perspective on daily interpersonal citizenship behaviors. J. Appl. Psychol. 100, 227–236. doi: 10.1037/a0038082 25314365

[B53] Voegtlin C. (2011). Development of a scale measuring discursive responsible leadership. J. Bus. Ethics 98, 57–73. doi: 10.1007/s10551-011-1020-9

[B54] Waqas M. Yahya F. Ahmed A. Rasool Y. Hongbo L. (2021). Unlocking employee's green behavior in fertilizer industry: the role of green HRM practices and psychological ownership. Int. Food Agribus. Manag. Rev. 24, 827–844. doi: 10.22434/IFAMR2020.0109

[B55] Wu C.-H. Parker S. K. (2017). The role of leader support in facilitating proactive work behavior: a perspective from attachment theory. J. Manage. 43, 1025–1049. doi: 10.1177/0149206314544745

[B56] Xie B. Xia M. Xin Y. Zhou W. (2016). Linking calling to work engagement and subjective career success: the perspective of career construction theory. J. Vocat. Behav. 94, 70–78. doi: 10.1016/j.jvb.2016.02.011

[B57] Xie B. Xu J. Fan J. Long L. Litchfield R. (2026). How do career shocks shape occupational calling? Testing a moderated chained mediation model. J. Appl. Psychol. 111, 468–487. doi: 10.1037/apl0001331 41213570

[B58] Zhang M. Shi H. Williams L. . (2023). An empirical test of the influence of rural leadership on the willingness to participate in public affairs from the perspective of leadership identification. Agriculture 13:1976. doi: 10.3390/agriculture13101976

[B59] Zhang Y. Puterman M. L. Nelson M. Atkins D. (2012). A simulation optimization approach to long-term care capacity planning. Oper. Res. 60, 249–261. doi: 10.1287/opre.1110.1026

[B60] Zhou J. George J. M. (2001). When job dissatisfaction leads to creativity: encouraging the expression of voice. Acad. Manag. J. 44, 682–696. doi: 10.2307/3069410

